# Deep Eutectic Solvent Micro-Functionalized Graphene Assisted Dispersive Micro Solid-Phase Extraction of Pyrethroid Insecticides in Natural Products

**DOI:** 10.3389/fchem.2019.00594

**Published:** 2019-08-23

**Authors:** Xiaoyu Song, Rui Zhang, Tian Xie, Shuling Wang, Jun Cao

**Affiliations:** ^1^Medical College, Hangzhou Normal University, Hangzhou, China; ^2^College of Material Chemistry and Chemical Engineering, Hangzhou Normal University, Hangzhou, China

**Keywords:** deep eutectic solvent, graphene, dispersive micro solid-phase extraction, pyrethroid insecticides, beebread, *Curcuma wenyujin* and *Dendrobium officinale*, ultra-high performance liquid chromatography

## Abstract

Deep eutectic solvent micro-functionalized graphene (DES-G) was synthesized and first applied as the adsorbent of dispersive micro solid-phase extraction (DMSPE) to extract five pyrethroid insecticides. In DMSPE, the target analytes were absorbed by DES-G and then desorbed by trace eluent, next, the treated samples were quantified via ultra-high performance liquid chromatography equipped with diode-array detection. A scanning electron microscope, transmission electron microscopy and Fourier transform infrared spectrometer were used to characterize the prepared DES-G. Furthermore, this method was verified under the selected conditions with the precision for retention times ranging from 0.43 to 0.57%, and repeatability ranged from 0.04 to 2.41% for peak areas. The developed method was successfully applied to determine pyrethroid insecticides residues in beebread, *Curcuma wenyujin* and *Dendrobium officinale* with the recoveries in the range of 80.9–114.1%.

## Introduction

Graphene, similar to graphite, C_60_ and carbon nanotubes in chemical structure composed of sp^2^ hybrid carbon atoms, is a new carbon nanomaterial. It contains a two-dimensional (2D) single layer carbon sheet structure stacked by hexagonal honeycomb lattices (Liu et al., 2012). In recent years, graphene has been used as adsorbent due to its large surface area and ease of adsorbing multiple compounds (Geim, 2009; Chandra et al., 2010; Li et al., 2011; Zhao et al., 2011). However, the main disadvantage tending to agglomerate leads to the instability of graphene in aqueous phase and limits its wider application (Stankovich et al., 2006a). The agglomeration is caused by the effects of π-conjugated and van der Waals forces, so it is able to solve this problem through the functional modification of graphene (Hayyan et al., 2015). To date, a variety of materials have been used to functionally modify graphene, such as polyoxometalates (Camille and Bandosz, 2009; Zhou and Han, 2010), organic diazonium salts (Englert et al., 2011), poly (oxyalkylene) amines (Hsiao et al., 2010), ionic liquids (ILs) (Zhao et al., 2014), deep eutectic solvent (DES) and so on. DES, a new green solvent, is a two-component or three-component eutectic mixture consisting of hydrogen bond acceptors (HBA) and hydrogen bond donors (HBD) in a suitable stoichiometric ratio. Choline chloride and betaine are commonly used as HBA of DES, while urea, polyols, and sugars are often used as HBD (Shishov et al., 2017). Owing to the low melting point, low toxicity, ease of synthesis, DES has been used to modify some materials such as cotton (Karimi et al., 2017), magnetic nanoparticles (Karimi et al., 2016) and grapheme, and then applied as the sorbents in the micro-extraction methods. In particular, the DES functionalized graphene (DES-G) has good dispersibility and stability in the aqueous phase, and has enormous potential application in the field of analytical chemistry (Radosevic et al., 2015).

The direct determination of trace analytes is usually limited due to the low analyte contents and complicated sample matrix, so the sample pretreatment is extremely necessary to extract target compounds from various complex samplesNowadays, the sample extraction methods including solid-phase extraction (SPE) (Yilmaz and Soylak, 2016; Yilmaz et al., 2018), pipette-tip solid-phase extraction (PT-SPE) (Wang et al., 2014), headspace solid phase micro-extraction (HS-SPME) (Farhadi et al., 2014), Quick Easy Cheap Effective Rugged Safe (QuEChERS) method (Zheng et al., 2013), liquid-phase microextraction (LPME), hollow fiber-based liquid-phase microextraction (HF-LPME), single-drop microextraction (SDME) and dispersive micro solid-phase extraction (DMSPE) have been reported. Among them, DMSPE introduced by Tsai et al. (2009) has attracted the attention of researchers because of its low consumption of organic solvents, and simple sample pretreatment manipulation (Kocot et al., 2013; Zheng et al., 2013). The process of DMSPE includes the sorption of compounds on adsorbent and following desorption by eluent at μL level. Therefore, the core of DMSPE is the choice of adsorbent. However, the use of DES-G as an adsorbent in DMSPE, as far as is known, has not been reported.

Food safety has always been a serious and controversial issue for the consumers, who paid close attention to the food safety-related events (Beulens et al., 2005). Pesticide residues and various additives have always been a hot topic in food safety. The use of pesticides such as organochlorine pesticides, organophosphorus pesticides, and pyrethroid insecticides, could control pests and increase crop yields. However, pesticides scattered in the air accumulated on the surface of crops and flowed into the food chain, causing serious damage to other species (Carvalho, 2006). Especially pyrethroids insecticides are more toxic for mammals due to their low water solubility, high liposolubility and ease of pass through biofilms (Feo et al., 2010). Therefore, some analytical methods were developed to extract and analyze pyrethroid insecticides in various complex matrices. For instance, Esteve-Turrillas et al. (2005) determined several pyrethroid residues in olive oils by SPE, in which the mixture of basic alumina and C_18_ was used as the adsorbent. Giroud et al. (2013) detected lambda-cyhalothrine, cypermethrine, deltamethrin, and esfenvalerate in beebread at trace level using the method of acetonitrile-based extraction. The improved QuEChERS method was proposed by Li et al. (2016) for the detection of pyrethroid insecticides in fruits and vegetables. Torbati et al. (2018) analyzed ten pyrethroids in apple and strawberry by homogeneous liquid-liquid microextraction. In order to improve the detection efficiency of pyrethroid insecticides and reduce organic solvent consumption, it is necessary to develop a green and efficient sample microextraction method.

In this work, several DES-Gs were synthesized by simple procedure and an optimum DES-G was selected as the adsorbent of DMSPE. Moreover, DES-G-based DMSPE method was investigated for the microextraction and determination of pyrethroid insecticides by ultra-high performance liquid chromatography (UHPLC). To confirm the reliability of this method, scanning electron microscope (SEM), transmission electron microscopy (TEM) and Fourier transform infrared spectrometer (FT-IR) were used for characterizing the functional changes of graphene. After a series of optimizations, the precision, repeatability, and linearity were studied under optimum conditions. Moreover, the proposed extraction approach was used for determining pesticide residues (fenpropathrin, ethofenprox, bifenthrin, fenvalerate, lambda-cyhalothrin) in natural products [beebread, *Curcuma wenyujin* and *Dendrobium officinale* (*D. officinale*)].

## Results and Conclusions

### FTIR, SEM, and TEM Analysis

**Figure 1:**
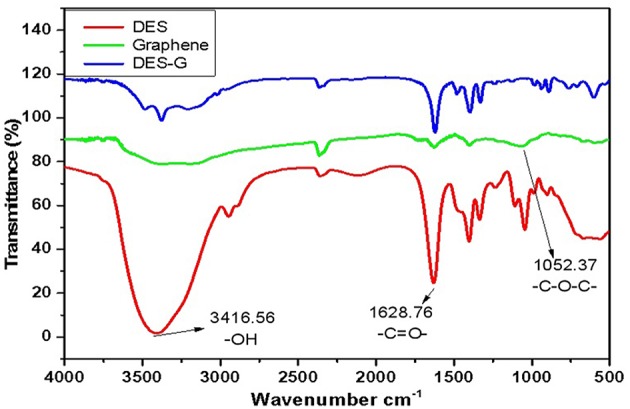
FTIR spectra of DES, Graphene, and DES-G.

FTIR, capable of qualitative and quantitative analysis of samples, was conducted to confirm the synthesis of DES and compare the changes before and after functionalization of graphene. Figure 1 shows the FTIR spectra of DES (DES 5), graphene (monolayer GO) and DES-G (DES 5-functionalized monolayer GO) as examples. As shown in the FTIR spectra of DES, the bonds of 3416.56 cm^−1^ and 1628.76 cm^−1^ represented −OH of betaine and −C = O− of glycerol, respectively. It is concluded that betaine and glycerol synthesized a new compound. Comparing the FTIR spectra of DES-G with DES and graphene, the −OH of DES and −C−O−C− (1052.37 cm^−1^) of graphene were obviously weakened.

**Figure 2:**
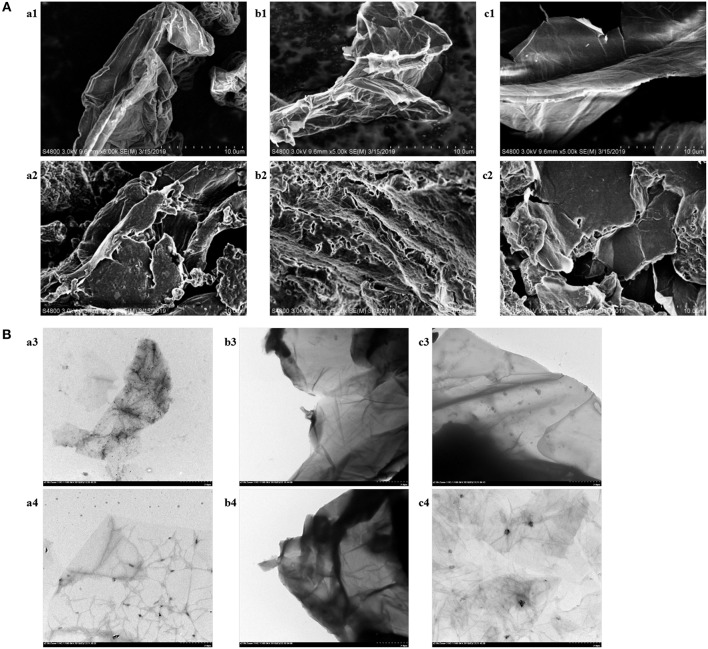
**(A)** SEM images of monolayer GO (a1), rGO-TEPA (b1), GO-COOH (c1),DES-monolayer GO (a2), DES-rGO-TEPA (b2), and DES-GO-COOH (c2). **(B)** TEM images of monolayer GO (a3), rGO-TEPA (b3), GO-COOH (c3),DES-monolayer GO (a4), DES- rGO-TEPA (b4), and DES-GO-COOH (c4).

In order to explore the influence of DES on the morphology, composition and internal structure of graphene, several samples were analyzed by SEM and TEM. The SEM and TEM images of three types of graphene were exhibited in Figure 2. For the longitudinal comparison of Figure 2A, considerable differences were observed in the morphology of the three functionalized graphenes (a2, b2, c2) compared to the original graphenes (a1, b1, c1). The loose stacking of functionalized graphenes, a structural deformation caused by the modification of DES, contributed to the dispersion of graphene in water and improved the stability of graphene in the aqueous phase (Hayyan et al., 2015). The sheet structure of graphene was clearly shown in the TEM images of Figure 2B. As can be seen from the pictures (a4, b4, c4), the functionalized graphene had a thinner sheet shape, which was consistent with the loose structure shown in the SEM images. The further horizontal comparison of SEM and TEM images was shown in the section of “Choice of the Type of Graphene.”

### Optimization of Extraction Conditions

The extraction efficiency was affected by various factors. The study selected several major factors to optimize extraction efficiency, starting with the initial conditions (1 μg/mL of sample solution, 2.5 μg/mL of graphene, shaking 2 min, 100 μL of methanol as the elution solvent).

### Choice of Deep Eutectic Solvent

**Figure 3:**
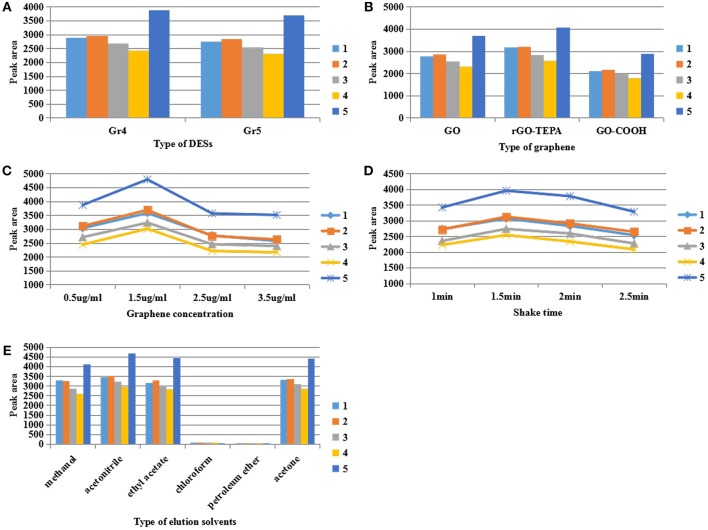
Effect of **(A)** the type of deep eutectic solvent, **(B)** the type of graphene, **(C)** graphene concentration, **(D)** shake time, **(E)** the type of elution solvent on the extraction of pyrethroid insecticide. The initial conditions were as follow: 1 μg/mL of sample solution, 2.5 μg/mL of monolayer GO, shaking 2 min, 100 μL of methanol as the elution solvent.

In order to select DES with good dispersion and suspension stability, six kinds of DES listed in Table 1 were prepared and five DES-Gs were synthesized under the same conditions. The DES1 (choline chloride:glucose = 1:1) was discarded because it was easy to solidify and difficult to mix uniformly with graphene. Then the five DES-Gs were prepared into relatively high concentrations (1,000 μg/mL), sealed and stood still. Figure S1 showed that all of the DES-Gs were dispersed homogeneously in water after sonication. However, the samples of Gr1 and Gr2 were precipitated rapidly only within 5 min and the Gr3 was precipitated gradually at 10 min. After standing for more than 48 h, Gr1, Gr2, and Gr3 were fully agglomerated and a clear water layer appeared. Meanwhile, Gr4 and Gr5 showed long-term stability. The results related to that the DES prepared by different mixtures had a discrepant dispersibility for graphene due to the different interaction of salts and HBDs, and the carboxyl functional group of betaine provided more oxygen atoms for forming hydrogen bonds with the HBD. So Gr4 and Gr5 were selected as the dispersant for extracting pesticide samples in subsequent dispersion studies. The result was shown in Figure 3A with the best extraction efficiency obtained by Gr4. This result was attributed to that the water-insoluble graphene was highly soluble in polyalcohol based-DES which had the least polarity (Zahrina et al., 2018). Consequently, the DES5 (Gr4) was selected for further research.

**Table 1:** Types and abbreviations of deep eutectic solvent.

**Salt**	**HBD^a^ type**	**Mole ratios of**	**Abbreviation**
		**salt:HBD**	**(DES^b^)**
Choline chloride	D-(+)-Glucose	1:1	DES1
	Propylene glycol + Water	1:1:1	DES2
	Glycerol	1:2	DES3
	Urea	1:2	DES4
Betaine	Glycerol	1:2	DES5
	Glycerol + Propylene glycol	1:1:1	DES6

a *HBD, hydrogen bond donors*.

b *DES, deep eutectic solvent*.

### Choice of the Type of Graphene

The structure of the base surface of graphene has a vital effect on its physicochemical properties such as the adsorption capability and solubility, which directly influence the concentration efficiency. To evaluate the effect of the type of graphene, monolayer GO, rGO-TEPA and GO-COOH were treated by DES5 under the same conditions. On the basis of the experimental results of Figure 3B, rGO-TEPA had the highest extraction efficiency for target analytes in comparison with monolayer GO and GO-COOH. That was because the base surface of monolayer GO mainly contained oxygen-containing functional groups, which changed the van der Waals force of the graphite molecules and affected their state in aqueous solution (Stankovich et al., 2006b). However, rGO-TEPA treated by DES began to precipitate at 30 min and showed visible delamination at 60 min (Figure S2). This phenomenon was likely attributed to the formation of intramolecular hydrogen bonds in rGO-TEPA, which competitively inhibited the interaction of graphene with DES, resulting in poor stability in water. It can be confirmed in Figure 2, the distinct sheet structures of functionalized rGO-TEPA that showed in the SEM images (b2) and its tightly stacked carbon layers resulted in a tendency to promote easy agglomeration. Additionally, no significant changes in the internal structure of functionalized rGO-TEPA (b4) compared to functionalized monolayer GO and GO-COOH (a4, c4) from the TEM images. The poor extraction efficiency of GO-COOH may be attributed that the carboxyl group of GO-COOH affected the adsorption of the target analytes by the graphene sheet. From Figure 3A, the surface of the GO-COOH before and after modification (c1, c2) was relatively smooth, without much continuous fold and wrinkle, resulting in the weak adsorption capacity. Therefore, considering the suspension stability and the extraction efficiency, monolayer GO was chosen for the next work.

### Effect of Graphene Concentration

Due to the unique two-dimensional nanosheet structure with the porous surface, graphene was used as adsorbent for indirect enrichment of target analytes (Liu et al., 2012). Therefore, the amount of graphene had an essential influence on the extraction efficiency. The effect of graphene was studied by changing its concentration of 0.5, 1.5, 2.5, and 3.5 μg/mL, respectively. From Figure 3C, the highest peak areas were achieved at the concentration of 1.5 μg/mL and the peak areas were significantly decreased when the concentration exceeded 1.5 μg/mL. This result was related to the adsorption saturation of the porous surface of graphene. It was well-known that the high concentration of graphene provided a large surface area and a large number of adsorption sites for the adsorption of the target analytes, thereby improving the concentration efficiency. However, the higher concentration of graphene produced stronger adsorption capability for the target compound that was difficult to be eluted by the eluent, resulting in poor concentration efficiency. So, in the following experiments, 1.5 μg/mL of monolayer GO was added to 5 mL of sample solution for adsorption.

### Effect of Extraction Time

In the course of preliminary experiment, it was found that the extraction time had an essential influence on the concentration efficiency. Thus, in the study, the effect of extraction time was investigated by using the oscillating shaker at the maximum speed (500 r) for 1.0, 1.5, 2.0, and 2.5 min. As shown in Figure 3D, the peak areas of the five pyrethroid insecticides increased to a maximum when the mixture was shaken for 1.5 min. Adequate extraction time ensured sufficient contact of the compounds of the sample solution with the surface of graphene, and improved the adsorption efficiency of graphene to the target analytes. However, it was observed that the response progressively decreased with the extraction time changing from 1.5 to 2.5 min. The result was ascribed to the enhancement of the interaction between analytes and graphene induced by extended extraction time, making the analytes difficult to elute by the eluent, thus resulting in the loss of analytes and decreased response. Therefore, the extraction time of 1.5 min was selected for the subsequent experiments.

### Choice of Elution Solvent

The type of elution solvents is a critical parameter that directly affected the concentration efficiency in the DMSPE. The elution solvents should be miscible with the target compounds and elute the compounds easily from the graphene in a small volume. In addition, the pyrethroid insecticides are lipophilic compounds (Serôdio and Nogueira, 2005). In order to find the suited eluent solvents for the analytes, methanol, acetonitrile, ethyl acetate, chloroform, petroleum ether and acetone were evaluated by comparing the response of the five compounds on the same condition (1 μg/mL of sample solution, 1.5 μg/mL of monolayer GO, shaking 1.5 min at 500 r, 100 μL elution solvent). The result is shown in Figure 3E. It was observed that acetonitrile showed a relatively high peak areas for five pyrethroid insecticides, followed by acetone, ethyl acetate and methanol, whereas chloroform and petroleum ether provided a poor response which was barely detectable. This phenomenon can be explained by the high elution ability of acetonitrile and the poor solubility of chloroform and petroleum ether to pesticides. Based on the above experimental results, acetonitrile was selected as the optimum elution solvent for further studies.

### Analytical Performance

The methodology of DES-G-based DMSPE was examined and evaluated with respect to linearity, precision, repeatability, the detection limit (LOD) and the quantitation limit (LOQ) under the above optimal conditions, 1.5 μg/mL of DES-G (as monolayer GO) used for adsorbing analytes, shaking 1.5 min at 500 r and 100 μL acetonitrile as the elution solvent. The linearity was investigated for the concentration of target compounds in the range of 0.01–1.0 μg/mL and the linear regression data was presented in Table 2. Analytical performance data of the target analytes was shown in Table 3. The precision, calculated on the basis of the relative standard deviations (RSDs, %), was used to verify the stability of the instrument. The intra-day precision of the presented method was studied by using six replicates in a day ranged from 0.43% to 0.57% for retention time, and the inter-day precision was obtained by injecting two needles at the same time for three consecutive days in the range of 0.43–0.56% for retention time. In order to verify the reliability of the proposed method, the repeatability was measured by three parallel sample preparation steps of the sample solution. The repeatability values, expressed as RSDs (%), varied between 0.04 and 2.41% for peak areas. The LODs were 0.016–0.24 ng/mL for five pyrethroid insecticides at signal-to-noise ratio of 3 and the LOQs (S/N ratio of 10:1) were 0.054–0.784 ng/mL.

**Table 2:** Linear regression data of the investigated analytes.

**Pyrethroid insecticide**	**Linear range (μg/mL)**	**Linear equation**	**Correlation coefficient (R^**2**^)**
Fenpropathrin	0.01–1	y = 3493.5 x – 87.577	0.9985
Ethofenprox	0.01–1	y = 3664.2 x – 2.166	0.9970
Bifenthrin	0.01–1	y = 3444.0 x + 15.584	0.9948
Fenvalerate	0.01–1	y = 3111.2 x – 50.736	0.9979
Lambda-cyhalothrin	0.01–1	y = 4790.3 x + 35.660	0.9956

*Conditions: 1 μg/mL of sample solution, 1.5 μg/mL of monolayer graphene oxide, shaking 1.5 min, 100 μL of acetonitrile as the elution solvent*.

**Table 3:** Analytical performance data of the investigated analytes.

**Pyrethroid insecticide**	**Intra-day precision**^a^ **(RSD%**, ***n*** **=** **6)**		**Inter-day precision**^a^ **(RSD%**, ***n*** **=** **6)**		**Repeatability**^a^** (RSD%**, ***n*** **=** **3)**		**LOD^b^ (ng/mL)**	**LOQ^b^ (ng/mL)**
	**Retention time**	**Peak area**	**Retention time**	**Peak area**	**Retention time**	**Peak area**		
Fenpropathrin	0.571	0.442	0.560	1.847	0.039	2.220	0.045	0.151
Ethofenprox	0.514	0.433	0.502	2.751	0.048	2.406	0.067	0.223
Bifenthrin	0.538	0.439	0.527	3.129	0.046	1.951	0.240	0.784
Fenvalerate	0.501	0.457	0.492	2.556	0.051	1.403	0.110	0.381
Lambda-cyhalothrin	0.429	0.533	0.425	3.157	0.056	2.305	0.016	0.054

a *Precision and repeatability are defined as the RSD (%)*.

b *LOD and LOQ are calculated on the basis of the signal-to-noise ratio of 3 and 10, respectively*.

### Analytical Application

**Figure 4:**
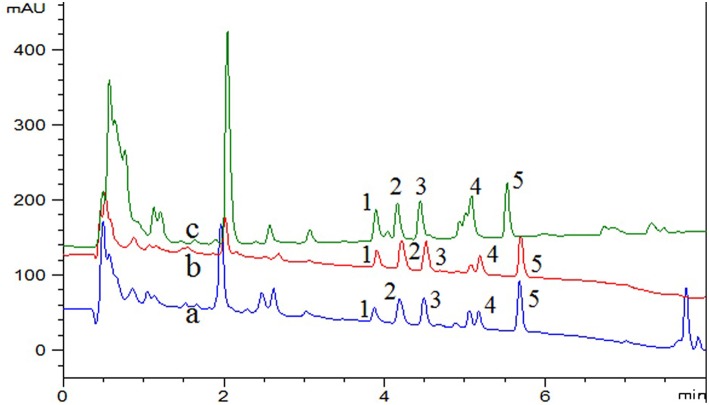
UHPLC-UV chromatograms of (a) beebread, (b) *Curcuma wenyujin*, (c) *Dendrobium officinale*, spiked with 0.05 μg/mL mixed standard of five pyrethroid insecticides. 1, fenpropathrin; 2, ethofenprox; 3, bifenthrin; 4, fenvalerate; 5, lambda-cyhalothrin.

The real samples, including beebread, *Curcuma wenyujin* and *D. officinale* were determined and analyzed to examine the availability of the developed method. The experimental results of typical chromatograms displayed in Figure 4. In order to further study the effect of the sample matrix on the detection sensitivity of the method, the spiked recovery was studied by spiking three levels of concentration (0, 0.05, and 0.5 μg/mL) of standard solution in real samples and the results were summarized in Table 4. The recoveries for beebread, *Curcuma wenyujin* and *D. officinale* were 80.9–112.9, 81.5–111.1, and 86.4–114.1%, respectively. The enrichment factor, as calculated by comparing the response of five compounds before and after the DES-G-based DMSPE process, was 46–59-folds (the concentration effect was shown in Figure S3). In addition, LC-MS/MS was performed to define the target analytes of real samples and the parameters were listed in Table 4.

**Table 4:** Determination of five pyrethroid insecticides in spiked samples.

**Analyte**	**Added (μg/mL)**	**Beebread**		***Curcuma wenyujin***		***Dendrobium officinale***		**M**	**M+23**
		**Found (μg/mL)**	**Recovery^a^ (%)**	**Found (μg/mL)**	**Recovery^a^ (%)**	**Found (μg/mL)**	**Recovery^a^ (%)**		
Fenpropathrin	0	n.d.^b^	–	n.d.^b^	–	n.d.^b^	–	–	372.43
	0.05	0.0536	107.1	0.0459	91.9	0.0570	114.1		
	0.5	0.5019	100.4	0.5465	109.3	0.4320	86.4		
Ethofenprox	0	n.d.^b^	–	0.0094	–	n.d.^b^	–	–	399.49
	0.05	0.0451	90.3	0.0417	83.4	0.0535	106.9		
	0.5	0.5351	107.0	0.5138	102.8	0.4957	99.1		
Bifenthrin	0	n.d.^b^	–	n.d.^b^	–	n.d.^b^	–	–	445.86
	0.05	0.0436	87.3	0.0407	81.5	0.0563	112.6		
	0.5	0.5398	108.0	0.5503	110.1	0.5125	102.5		
Fenvalerate	0	n.d.^b^	–	n.d.^b^	–	n.d.^b^	–	–	442.90
	0.05	0.0457	91.4	0.0467	93.3	0.0542	108.5		
	0.5	0.4856	97.1	0.5555	111.1	0.4868	97.4		
Lambda–cyhalothrin	0	n.d.^b^	–	n.d.^b^	–	n.d.^b^	–	449.85	–
	0.05	0.0404	80.9	0.0490	98.0	0.0606	101.2		
	0.5	0.5643	112.9	0.5262	105.2	0.5034	100.7		

a *Recovery (%) = (the amount found in the spiked sample – the amount found in the sample) × 100 / the amount added*.

b *n.d., not detectable*.

### Comparison of DES-G-Based DMSPE With Other Methods

The proposed method was compared with other extraction methods that applied for the determination of pyrethroid insecticides. The characteristics of developed method and the comparison of the amount of adsorbent, the type and amount of extraction solvent, extraction time, RSD (%) and LOD were listed in Table 5. It can be seen that the LODs of this method were lower than those achieved by SPE (Esteve-Turrillas et al., 2005), LLE-DLLME (Farajzadeh et al., 2014), and MDSPE (Badawy et al., 2018). In comparison with other methods, the amounts of adsorbent and extraction solvent were obviously reduced: only 1.5 μg/mL of DES-G and 100 μL of acetonitrile. Furthermore, the extraction time of only 1.5 min significantly improved the extraction efficiency. It is worth mentioning that the RSD (%) was relatively low, which proved the stability of the method. Thus, DES-G-based DMSPE combined with UHPLC-DAD was rapid, stable, sensitive and environmentally friendly for the determination of pyrethroid insecticides in natural products.

**Table 5:** Comparison of DES-G based DMSPE method with other methods for the analytes of pyrethroid insecticides.

**Sample**	**Extraction method^a^**	**Analysis technique**	**Adsorbent^b^**	**Extraction solvent^c^**	**Extraction time**	**Linear range**	**Repeatability (RSD, %)**	**LOD**	**References**
Vegetable oils	SPE	GC-MS-MS	C_18_ (500 mg) and basic alumina (2 g)	acetonitrile (10 mL)	40 min	10–1000 μg/L	4–13%	0.3–1.4 ng/g	Tsai et al., 2009
Beebread	acetonitrile-based extraction	UHPLC-MS	PSA (150 mg)	heptane (5 mL)	–	0.1–10 ng/g	<20%	0.013–0.100 ng/g	Kocot et al., 2013
Fruit juices	SPI-SFO-HLLME	GC-MS	–	pivalic acid (260 μL)	5 min	0.023–500 ng/mL	4–7%	0.006–0.034 ng/mL	Carvalho, 2006
Vegetable oil	LLE-DLLME	GC-FID	–	DMF (1.0 mL) and 1,1,2-trichloroethane (75 μL)	10 min	55–6000 ng/g	5–13%	20–170 ng/g	Torbati et al., 2018
Water	MDSPE	HPLC	Ch-Si MNPs (50/100/200 mg)	acetonitrile/methanol (1:1, 5 mL)	20 min	0.0125–0.15 μg/mL	–	0.002–0.046 μg/mL	Zahrina et al., 2018
Beebread and apple	DMSPE	UHPLC-DAD	DES-G (1.5 μg/mL)	acetonitrile (100 μL)	1.5 min	0.01–1.0 μg/mL	0.04–2.41%	0.016–0.24 ng/mL	This work

a *Extraction Method: SPE, solid-phases extraction; HLLME, homogeneous liquid–liquid microextraction; LLE-DLLME, liquid-liquid extraction-dispersive liquid-liquid microextraction; MDSPE, magnetic dispersive solid-phase extraction; DMSPE, dispersive micro solid-phase extraction*.

b *Adsorbent: PSA, primary and secondary amines; Ch-Si MNPs, chitosan-siloxane magnetic nanoparticles*.

c *Extraction Solvent: DEF, dimethylformamide*.

## Conclusions

The method of DES-G assisted DMSPE with UHPLC-DAD was developed for the trace analysis of five pyrethroid insecticides in beebread, *Curcuma wenyujin* and *Dendrobium officinale*. In this work, the DES, easy to be produced and low toxicity, was a new type of functional reagent and effectively modified the graphene. Moreover, the graphene, micro-functionalized by DES, not only retained the high adsorption capacity of graphene, but weakened its main defect that was prone to agglomeration. After the optimization of a series of parameters, under optimized conditions, the sensitivity, reliability and practicability of this method were verified with the LODs were 0.016–0.24 ng/mL, the repeatability was 0.04–2.41% and the recovery ranged from 80.9 to 112.9%. Based on the above experimental results, the method was simple, rapid, sensitive, efficient and environmentally friendly. In addition, the functionalization method of graphene and the application of DES-G are worthy of further exploration. There is no doubt that the future of graphene in the field of analytical chemistry is still very bright and is full of potential.

## Materials and Methods

### Reagents and Materials

Fenpropathrin, ethofenprox, bifenthrin, fenvalerate, lambda-cyhalothrin were produced by Shanghai Pesticide Research Institute Co., Ltd. (Shanghai, China). Trichloromethane and glycerol were purchased from Xilong Scientific Co., Ltd. (Shantou, China). Methanol (HPLC grade), acetonitrile (HPLC grade), ethyl acetate [analytical grade (AR)], petroleum ether (AR), acetone (AR), choline chloride (AR), and betaine (purity 98%) were provided by Sinopharm Chemical Reagent Co., Ltd. (Shanghai, China). D-(+)-Glucose was purchased from Shenggong Biological Engineering Co., Ltd. (Shanghai, China). Propylene glycol (99.8%) was purchased from Beijing Bailingwei Technology Co., Ltd. (Beijing, China). Ultrapure water was supplied by Hangzhou Wahaha Group Co., Ltd. (Hangzhou, China). Monolayer graphene oxide (GO) was purchased from Suzhou Carbon Graphene Technology Co., Ltd. (Suzhou, China). Reduced graphene oxide-tetraethylene pentamine (rGO-TEPA) and carboxyl functionalized graphene oxide (GO-COOH) were supplied by Nanjing Xianfeng Nano Material Technology Co., Ltd. (Nanjing, China). Beebread, *Curcuma wenyujin* and *D. officinale* were purchased from local market in Beijing (Beijing, China), Wenzhou (Wenzhou, China) and Zhuji (Zhuji, China), respectively. All stock solutions of pyrethroid insecticides were dissolved by methanol at 1,000 μg/mL and stored at 4°C. Working solutions were obtained by diluting the stock solution with ultrapure water.

### Instrumentation and Conditions

Agilent 1,290 series UHPLC system (Agilent Technologies Co., Ltd., USA) coupled with a high speed binary pump and a diode array detector (DAD) was used for the quantitative analysis of the pyrethroid insecticides. The separation of target analytes was carried out on Agilent SB-C_18_ column (2.1 × 100 mm, 1.8 μm). The detection wavelength was set at 210 nm. The mobile phases were (A) water and (B) acetonitrile with the gradient as follows: 70–90% (B) for 0–5 min, 90–100% (B) for 5–8 min and then a re-equilibration of 5 min. After being filtered through 0.22 μm filter, the sample solution of 2 μL was injected to UHPLC system and analyzed at 35°C with the flow rate at 0.4 mL/min. The SEM (Hitachi S4800, Tokyo, Japan), TEM (Hitachi HT-7700, Tokyo, Japan) and FT-IR (Thermo Scientific Nicolet iS5 spectrometer, Madison, USA) were used for the characterization of DES, graphene and DES-G.

### DES Preparation

All DESs were synthesized by mixing different salts and HBD listed in Table 1 according to previous works (Hayyan et al., 2015; Ruesgas-Ramón et al., 2017; Zahrina et al., 2018). The choline chloride and betaine were dried at 60°C for no more than 6 h under vacuum before utilization due to the hygroscopic nature. Then the mixture was added to a jacketed vessel and heated at 70°C while stirring at 300 r. The final liquid was transferred to a well-sealed glass bottle until a uniform transparent liquid was obtained. The fresh DES samples were used through all the work in order to avoid any differences in DES properties caused by temperature and humidity changes.

### Graphene Functionalization Process

The treatment of graphene functionalization was based on previous research (Hayyan et al., 2015). Seven milliliter of DES and 200 mg of graphene were separately weighed and placed in a beaker. The mixture was stirred at room temperature until it was completely mixed. After sealing, it was sonicated at 60°C for 3 h and then dried under vacuum at 100°C for 3 h. The appropriate DES-G was weighed and diluted with water to prepare a 1,000 μg/mL stock solution for subsequent use.

### Sample Preparation

A sample processing method of fresh fruits and vegetables reported by Li et al. (2016) was adopted for the preparation of the beebread with minor modifications. Briefly, 15 g of sample was weighed and 15 mL of acetonitrile were placed and mixed in a 50 mL centrifuge tube. Following full extraction, 1.50 g of NaCl and 6.00 g of anhydrous magnesium sulfate (MgSO_4_) were added and swirled for extraction and salting out until the solvent interacted sufficiently with the entire samples. The supernatant was transferred into a 15 mL centrifuge tube containing 2.4 g of C_18_, 1.16 g of MgSO_4_ and 0.04 g of graphitized carbon black after centrifugation for 10 min at 4,000 r. The mixture was swirled to mix thoroughly and then centrifuged for 5 min (4,000 r). Finally, the supernatant was decanted and stored in a glass bottle (4°C).

The sample treatment procedure of *Curcuma wenyujin* and *D. officinale* was based on the Chinese Pharmacopeia (2015 edition). The specific steps are as follows: A precisely weighed 3 g of *Curcuma wenyujin* powder (40 mesh) was placed in a 50 mL centrifuge tube and 15 mL of 1% glacial acetic acid solution was added. The mixture was vortexed to completely infiltrate and left for 30 min, then 15 mL of acetonitrile was added into the centrifuge tube and swirled on a vortex mixer to homogenizing the sample. After the mixture was vigorously oscillated by vortex for 5 min (500 r), 6.00 g of MgSO_4_ and 1.50 g of anhydrous sodium acetate were mixed with the sample, shook the mixture immediately and then oscillated (500 r, 3 min), cooled in ice bath (10 min), centrifuged (4,000 r, 5 min). Next, the supernatant of 9 mL was transferred to a 50 mL centrifuge tube containing 900 mg of MgSO4, 300 mg of N-propylethylenediamine (PSA), 300 mg of C_18_, 300 mg of silica gel, and 90 mg of graphitized carbon black. The mixture was swirled to mix thoroughly and was oscillated vigorously (500 r, 5min). After centrifugation for 5 min (4,000 r), 5 mL of the supernatant was accurately pipetted and stored in a glass bottle (4°C).

### Extraction and Analysis

**Figure 5:**
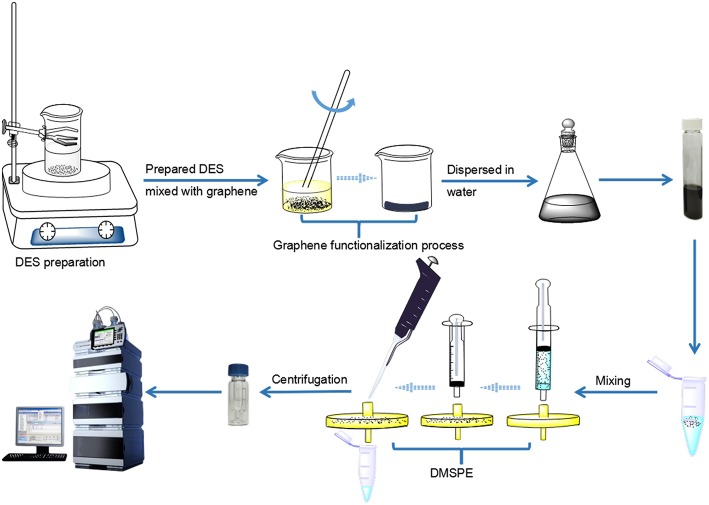
The brief procedure of DES-G assisted DMSPE combined with UHPLC-DAD method.

The sample solution and graphene were placed in a 25 mL centrifuge tube at a concentration ratio of 1:2.5. The mixture was shaken to make the compounds fully bind with graphene at 500 r for 2 min. After adsorption, the graphene-bound analytes were filtered by a 0.22 μm nylon filter and left in the filter head. Finally, the target analytes were separated from graphene by 100 μL of eluent, and the eluate was further centrifuged at 13,000 r (5 min) for UHPLC analysis. The brief procedure of this work was shown in Figure 5.

## Data Availability

The authors declare that all relevant data supporting the findings of this study are available within the article and Supplementary Information Files.

## Author Contributions

All authors listed have made a substantial, direct and intellectual contribution to the work, and approved it for publication.

### Conflict of Interest Statement

The authors declare that the research was conducted in the absence of any commercial or financial relationships that could be construed as a potential conflict of interest.
