# Supplementary material for: Deep Eutectic Solvent Micro-Functionalized Graphene Assisted Dispersive Micro Solid-Phase Extraction of Pyrethroid Insecticides in Natural Products
Source: Front Chem. 2019 Aug 23;7:594. doi: 10.3389/fchem.2019.00594 (PMC6716533; doi:10.3389/fchem.2019.00594)

**Deep eutectic solvent micro-functionalized graphene assisted dispersive micro solid-phase extraction of pyrethroid insecticides in natural products**

**Xiaoyu Song^1^, Rui Zhang^1^, Tian Xie^1*^, Shuling Wang^1*^, Jun Cao^1, 2*^**

^1^Medical College, Hangzhou Normal University, Hangzhou 311121, P.R. China

^2^College of Material Chemistry and Chemical Engineering, Hangzhou Normal University, Hangzhou 311121, P.R. China

**Fig. S1.** Prepared DES and suspension behavior of monolayer GO treated with DES 2 to DES 6 (Gr 1 to Gr 5) in distillated water.


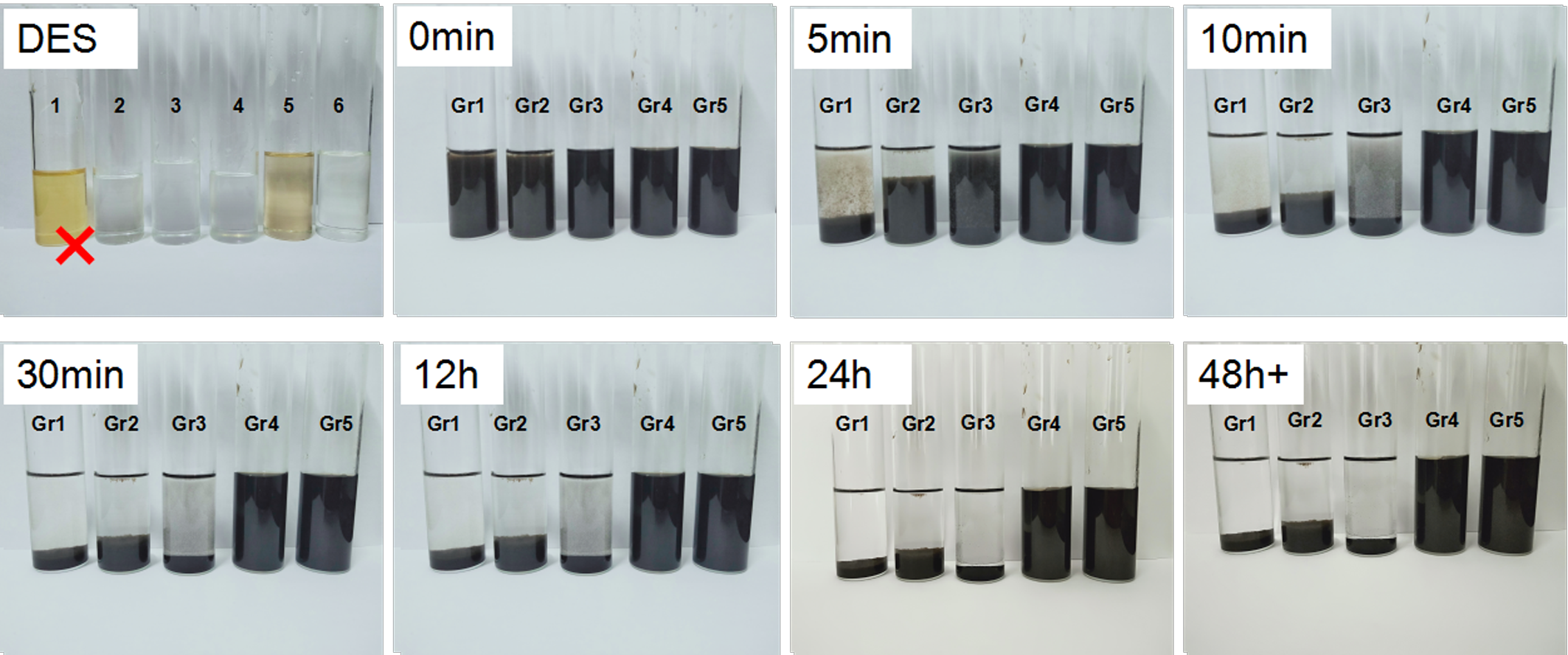


**Fig. S2.** Suspension behavior of three kinds of graphene treated with DES 5 in distillated water. 1. Monolayer GO; 2. rGO-TEPA; 3. GO-COOH.


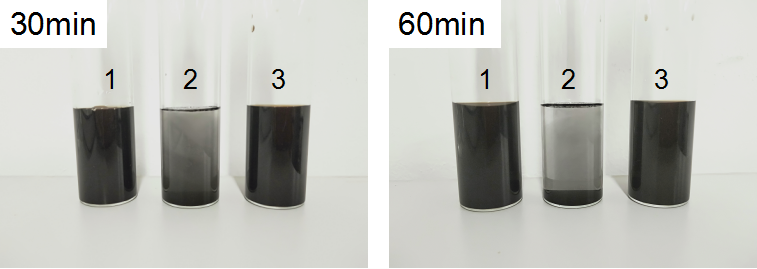


**Fig. S3.** UHPLC-UV chromatograms of five pyrethroid insecticides before and after DES-G extraction. 1. fenpropathrin, 2. ethofenprox, 3. bifenthrin, 4. fenvalerate, 5. lambda-cyhalothrin.


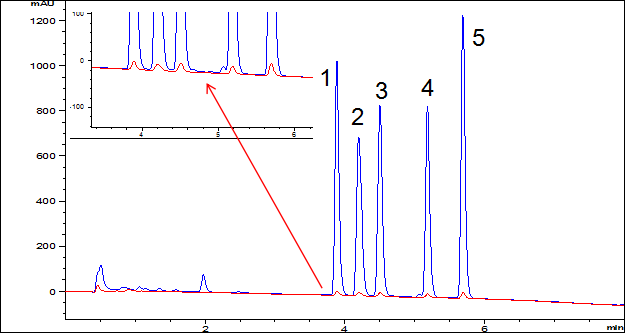

Supplement: Supplementary file 1 [file Table_1.doc]
